# The complete mitochondrial genome of *Poecilia formosa* (*Poecilia,* Cyprinodontidae) and phylogenetic studies of cyprinodontiformes

**DOI:** 10.1080/23802359.2019.1681308

**Published:** 2019-10-30

**Authors:** Youkun Huang, Bingjian Liu, Fang Meng, Qi Wang, Kehua Zhu, Jianshe Zhang, Fei Jing, Liping Xia, Yifan Liu

**Affiliations:** a National Engineering Research Center for Marine Aquaculture, Zhejiang Ocean University, Zhoushan, China;; b National Engineering Laboratory of Marine Germplasm Resources Exploration and Utilization, Zhejiang Ocean University, Zhoushan, China

**Keywords:** *Poecilia formosa*, mitochondrial genome, evolutionary relationships

## Abstract

We report the complete mitochondrial genome sequence of *Poecilia formosa*. The genome is found to be 16636 bp in length and has a base composition of A (29.59%), G (14.61%), C (28.26%), and T (27.54%). Similar to other *Poecilia* species, it contains a typically conserved structure including 13 protein-coding genes, 2 rRNA genes, 1 control region (D-loop), and 22tRNA genes. The proportion of coding sequences with a total length of 11,533 bp is 69.33%, which encodes 3837 amino acids. All protein-coding genes started with Met, ND1, CO1, ATP8, ATP6, CO3, ND4L, ND5, ND6 and CytB ended by TAA as a stop codon, ND2 and ND3 ended by TAG as a stop codon, CO3 and ND4 ended by a single T. The lengths of 12S ribosomal RNA is 948 bp, ranging from 70 bp to 1018 bp, and the lengths of 16S ribosomal RNA is 1674 bp, ranging from 1090 bp to 2764 bp. The length of control region is 879 bp, ranging from 15757 bp to 16636 bp, respectively. The complete mitochondrial genome sequence provided here would be useful for further understanding the evolution of ratite and conservation genetics of *Poecilia formosa.*

*Poecilia formosa*, belongs to the family *Poecilia*, Cyprinodontidae, is a freshwater aquarium fish which distributes in the North America, Texas, and northern Mexico, Nueces River and Grande River (Schlupp et al. 2002). Although two complete mitochondrial (mt) genomes belonging to *Poecilia formosa* have been determined, the *Poecilia formosa* mt genome sequence has not been reported yet.

Here, we sequenced and characterised the complete mt genome of *Poecilia formosa*. The specimen was collected from the part of Nueces River between Three Rivers and George West (36°26′11″N, 118°49′24″W). Then it stored in a refrigerator of −80 °C in Zhejiang Engineering Research Centre for Mariculture and Fishery Enhancement Museum(Accession number PF180513). Total genomic DNA was extracted from muscle tissue of individual using the phenol-chloroform method (Barnett and Larson 2012). The calculation of base composition and phylogenetic construction were conducted by MEGA6.0 software (Tamura et al. 2013). The transfer RNA (tRNA) genes were generated using the programme tRNAs-can-SE (Lowe and Eddy 1997). The mitochondrial genome sequence of *Poecilia formosa* with the annotated genes was deposited in GenBank with the accession number of KT715811.

Similar to the typical mitogenome of vertebrates, the mitogenome of *Poecilia formosa* is a closed double-stranded circular molecule of 16636 nucleotides (GenBank accession No. KT715811), which contains 13 protein-coding genes, 2 ribosomal RNA genes, 22 tRNA genes and 2 main non-coding regions (Boore 1999; Zhu et al., 2018a; Zhu et al., 2018b). The contents of A, G, T and C are 29.59%, 14.61%, 27.54%, and 28.26%, respectively. Most mitochondrial genes are encoded on H-strand except for ND6 and eight tRNA genes (Gln, Ala, Asn, Cys, Tyr, Ser, Glu and Pro), which are encoded on the L-strand. The proportion of coding sequences with a total length of 11,533 bp is 69.33%, 13 protein-coding (PCGs) genes encode 3837 amino acids in total. All protein-coding genes started with Met, which is quite common in vertebrate mtDNA (Miya et al. 2001; Liu et al. 2017). ND1, CO1, ATP8, ATP6, CO3, ND4L, ND5, ND6 and CytB ended by TAA as a stop codon, ND2 and ND3 ended by TAG as a stop codon, CO3 and ND4 ended by a single T. The 12S rRNA and 16S rRNA are 948 and 1674 bp, which are both located in the typical positions between tRNA-Phe and tRNA-Leu (UUA), separated by tRNA-Val. The origin of light-strand replication is located in a cluster of five tRNA genes (WANCY) as in other vertebrates (Petrillo et al. 2006), The length of control region is 879 bp, ranging from 15757 bp to 16636 bp.

Phylogenetic analysis included mt genome of *Poecilia formosa* and the other 13 species that are from the order *Kryptolebias*, *Poeciliopsi*s, *Rivulus*, *Xiphophorus*, *Poecilia*, *Orestias*, *Cyprinodon* and *Austrolebias,* which belong to Cyprinodontiformes. The results of the present study support that *Poecilia formosa* has a closest relationship with *Poecilia reticulata and Poecilia sphenops* (Figure 1).

**Figure 1.**
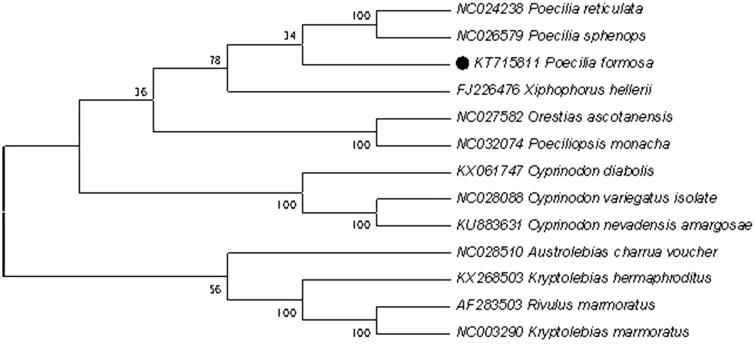
Phylogenetic tree derived from NJ based on concatenated nucleotide sequences of twelve PCGs. Including Poecilia reticulata, Poecilia sphenops, Poecilia formosa, Poeciliopsis monacha, Xiphophorus hellerii, Orestias ascotanensis, Cyprinodon diabolis, Cyprinodon veriegatus isolate, Cyprinodon nevadensis amargosae, Austrolebias charrua voucher, Kryptolebias hemaphroditus, Rivulus marmoratus,.Kryptolebias marmoratus.

## References

[CIT0001] Barnett R, Larson G 2012 A phenol-chloroform protocol for extracting DNA from ancient samples. Method Mol Biol. 840:13–19. 10.1007/978-1-61779-516-9_2 22237516

[CIT0002] Boore JL 1999 Animal mitochondrial genomes. Nucleic Acids Res. 27:1767–1780. 10101183 10.1093/nar/27.8.1767 PMC148383

[CIT0003] Liu J, Ding Q, Gao L 2017 The complete mitochondrial genome of North Island brown kiwi (*Apteryx mantelli*). Mitochondrial DNA Part B. Issue 1. 10.1080/23802359.2016.1186511 PMC7800362 33490432

[CIT0004] Lowe T, Eddy S 1997 tRNAscan-SE: a program for improved detection of transfer RNA genes in genomic sequence. Nucleic Acids Res. 25:955–964. 9023104 10.1093/nar/25.5.955 PMC146525

[CIT0005] Miya M, Kawaguchi A, Nishida M 2001 A case study for Moderate-Scale evolutionary genomics with 38 newly determined complete mitochondrial DNA sequences. Mol Biol Evol. 18:1993–2009. 11606696 10.1093/oxfordjournals.molbev.a003741

[CIT0006] Petrillo M, Silvestro G, Nocera PPD, Boccia A, Paolella G 2006 Stem-loop structures in prokaryotic genomes. BMC Genomics. 7:170. 16820051 10.1186/1471-2164-7-170 PMC1590033

[CIT0007] Schlupp I, Parzefall J, Schartl M 2002 Biogeography of the amazon molly, Poecilia formosa[J]. J Biogeogr. 29:1–6.

[CIT0008] Tamura K, Stecher G, Peterson D, Filipski A, Kumar S 2013 MEGA6: molecular evolutionary genetics analysis version 6.0. Mol Biol Evol. 30:2725–2729. 24132122 10.1093/molbev/mst197 PMC3840312

[CIT0009] Zhu K, Gong L, Jiang L, Liu L, Lü Z, Liu B-J 2018a Phylogenetic analysis of the complete mitochondrial genome of *Anguilla japonica* (Anguilliformes, Anguillidae). Mitochondrial DNA Part B. 3:536–537. 10.1080/23802359.2018.1467225 PMC7799773 33474232

[CIT0010] Zhu K, Lü Z, Liu L, Gong L, Liu B 2018b The complete mitochondrial genome of *Trachidermus fasciatus* (Scorpaeniformes: Cottidae) and phylogenetic studies of Cottidae. Mitochondrial DNA Part B. 3:301–302. 10.1080/23802359.2018.1445480 PMC7799978 33474152

